# Crystal structure, Hirshfeld surface analysis and inter­action energy and DFT studies of (*S*)-10-propargyl­pyrrolo­[2,1-*c*][1,4]benzodiazepine-5,11-dione

**DOI:** 10.1107/S2056989020002698

**Published:** 2020-03-03

**Authors:** Dounia Jeroundi, Ahmed Mazzah, Tuncer Hökelek, El Mestafa El Hadrami, Catherine Renard, Amal Haoudi, El Mokhtar Essassi

**Affiliations:** a Laboratory of Applied Organic Chemistry, Sidi Mohamed Ben Abdellah University, Faculty of Sciences and Techniques, Road Immouzer, BP 2202 Fez, Morocco; b USR 3290 Miniaturisation pour l’analyse, la synthèse et la protéomique, 59655, Villeneuve d’Ascq Cedex, Université Lille1, France; c Department of Physics, Hacettepe University, 06800 Beytepe, Ankara, Turkey; d Unité de Catalyse et de Chimie du Solide (UCCS), UMR 8181, Ecole Nationale Supérieure de Chimie de Lille, Université Lille 1, 59650 Villeneuve d’Ascq Cedex, France; e Laboratoire de Chimie Organique Hétérocyclique URAC 21, Pôle de Compétence Pharmacochimie, Av. Ibn Battouta, BP 1014, Faculté des Sciences, Université Mohammed V, Rabat, Morocco

**Keywords:** crystal structure, benzodiazepine, pyrrole, Hirshfeld surface

## Abstract

The title compound, C_15_H_14_N_2_O_2_, consists of pyrrole and benzodiazepine units linked to a propargyl moiety, where the pyrrole and diazepine rings adopt half-chair and boat conformations, respectively. The absolute configuration was assigned on the the basis of l-proline, which was used in the synthesis of benzodiazepine. In the crystal, weak C—H_Bnz_⋯O_Diazp_ and C—H_Proprg_⋯O_Diazp_ (Bnz = benzene, Diazp = diazepine and Proprg = proparg­yl) hydrogen bonds link the mol­ecules into two-dimensional networks parallel to the *bc* plane, enclosing *R*_4_^4^(28) ring motifs, with the networks forming oblique stacks along the *a*-axis direction. The Hirshfeld surface analysis of the crystal structure indicates that the most important contributions for the crystal packing are from H⋯H (49.8%), H⋯C/C⋯H (25.7%) and H⋯O/O⋯H (20.1%) inter­actions. Hydrogen bonding and van der Waals inter­actions are the dominant inter­actions in the crystal packing. Computational chemistry indicates that in the crystal, C—H⋯O hydrogen-bond energies are 38.8 (for C—H_Bnz_⋯O_Diazp_) and 27.1 (for C—H_Proprg_⋯O_Diazp_) kJ mol^−1^. Density functional theory (DFT) optimized structures at the B3LYP/6–311 G(d,p) level are compared with the experimentally determined mol­ecular structure in the solid state. The HOMO–LUMO behaviour was elucidated to determine the energy gap.

## Abstract 2

The title compound consists of pyrrole and benzodiazepine units linked to a propargyl moiety, where the pyrrole and diazepine rings adopt half-chair and boat conformations, respectively. In the crystal, weak C—H_Bnz_⋯O_Diazp_ and C—H_Proprg_⋯O_Diazp_ (Bnz = benzene, Diazp = diazepine and Proprg = proparg­yl) hydrogen bonds link the mol­ecules into two-dimensional networks parallel to the *bc* plane, enclosing 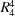
(28) ring motifs.

## Chemical context   

Over the past few decades, compounds bearing heterocyclic nuclei have received much attention of chemists and biologists because of their importance in the development of chemotherapeutic agents and a wide variety of drugs (Cargill *et al.*, 1974 ▸; Micale *et al.*, 2004 ▸; Hadac *et al.*, 2006 ▸; Ourahou *et al.*, 2011 ▸). 1,4-Benzodiazepines and their derivatives have attracted the attention of chemists since the early 1960s, mainly because of the broad spectrum of biological properties exhibited by this class of compounds, in particular their psychopharmacological properties (Thurston & Langley, 1986 ▸; Kamal *et al.*, 2007 ▸; Antonow *et al.*, 2007 ▸; Archer & Sternbach, 1968 ▸; Mohiuddin *et al.*, 1986 ▸, Bose *et al.*, 1992 ▸; Gregson *et al.*, 2004 ▸). The vast commercial success of these medicinal agents has resulted in their chemistry being a major focus of research in the field of medicinal chemistry and many such ring systems having been described (Benzeid *et al.*, 2009*a* ▸,*b* ▸; Randles & Storr, 1984 ▸; Sugasawa *et al.*, 1985 ▸; Cipolla *et al.*, 2009 ▸). Pyrrolo­[2,1-*c*][1,4]benzodiazepines are a group of potent chemicals produced by Streptomyces species. For their anti­cancer activity, see: Bose *et al.* (1992 ▸); Cargill *et al.* (1974 ▸); Gregson *et al.* (2004 ▸).
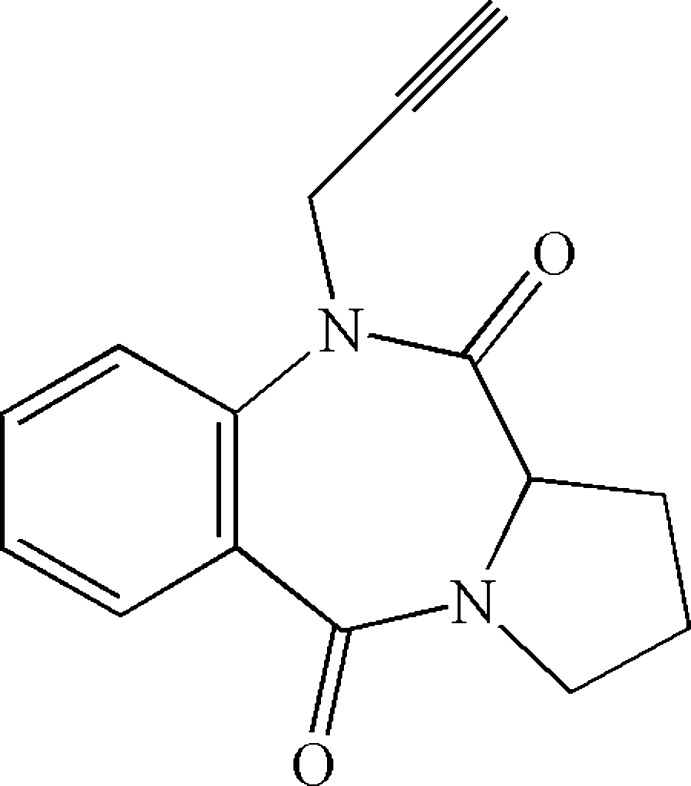


**Figure 1:**
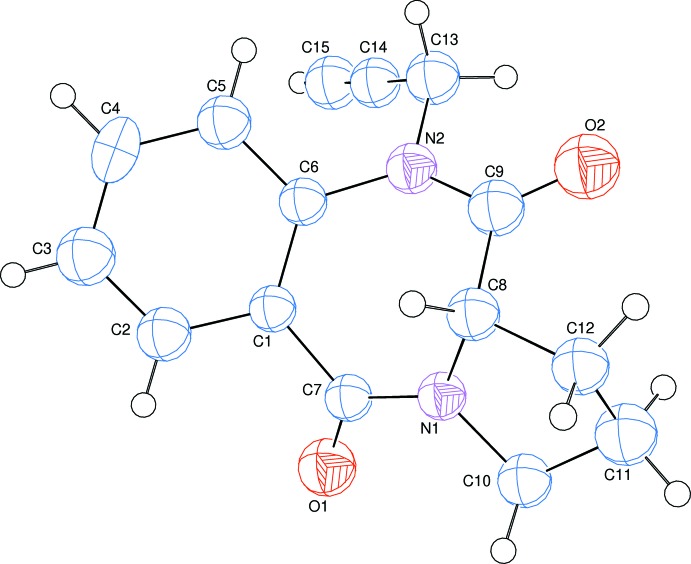
The mol­ecular structure of the title compound with the atom-numbering scheme. Displacement ellipsoids are drawn at the 50% probability level.

In a continuation of our research work on the advancement of benzodiazepine derivatives, we have developed a new synthethis for 10-propargyl­pyrrolo­[2,1-*c*][1,4]benzodiazepine-5,11-dione (Fig. 1 ▸) in good yield from pyrrolo­[2,1-*c*][1,4]benzodiazepine with propargylbromide in the presence of tetra-*n*-butyl­ammonium bromide (TBAB) as catalyst and potassium carbonate as base (Makosza & Jonczyk, 1976 ▸). The synthesized compound was characterized by single-crystal X-ray diffraction as well as Hirshfeld surface analysis. The results of the calculations by density functional theory (DFT), carried out at the B3LYP/6-311G (d,p) level, are compared with the experimentally determined mol­ecular structure in the solid state.

## Structural commentary   

The title compound, (I), consists of pyrrole and benzodiazepine units linked to a propargyl moiety (Fig. 1 ▸). The five-membered pyrrole ring (N1/C8/C10–C12) adopts a half-chair conformation [puckering parameters *q*_2_ = 0.376 (3) Å and θ = 94.4 (4)°] while the seven-membered diazepine ring (N1/N2/C1/C6–C9) adopts a boat conformation [*Q*_T_ = 0.9262 (13), *q*_2_ = 0.9070 (14), *q*_3_ = 0.1875 (16) Å, φ_2_ = 105.6 (4) and φ = 161.4 (5)°]. In the propargyl moiety, the N2—C13—C14 and C13—C14—C15 bond angles are 112.66 (17)° and 177.4 (3)°, respectively.

## Supra­molecular features   

**Figure 2:**
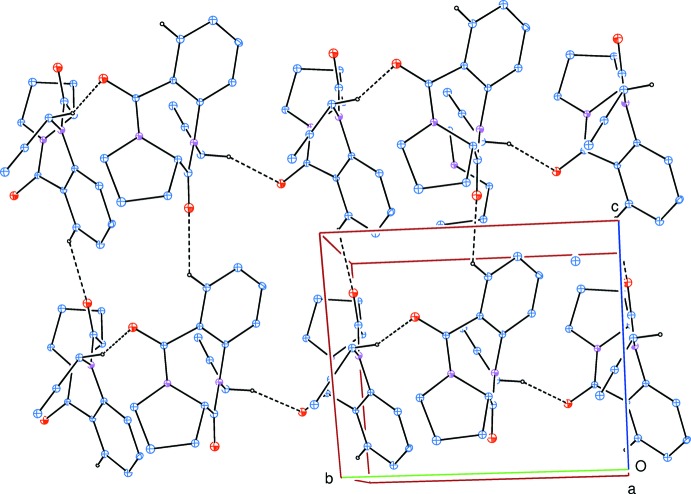
A partial packing diagram viewed along the *a*-axis direction with weak inter­molecular C—H_Bnz_⋯O_Diazp_ and C—H_Proprg_⋯O_Diazp_ (Bnz = benzene, Diazp = diazepine and Proprg = proparg­yl) hydrogen bonds (dashed lines). H atoms not included in hydrogen bonding have been omitted for clarity.

In the crystal, weak C—H_Bnz_⋯O_Diazp_ and C—H_Proprg_⋯O_Diazp_ (Bnz = benzene, Diazp = diazepine and Proprg = proparg­yl) hydrogen bonds (Table 1 ▸) link the mol­ecules into two-dimensional networks parallel to the *bc* plane, enclosing 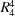
(28) ring motifs (Fig. 2 ▸), with the networks forming oblique stacks along the *a*-axis direction.

## Hirshfeld surface analysis   

**Figure 3:**
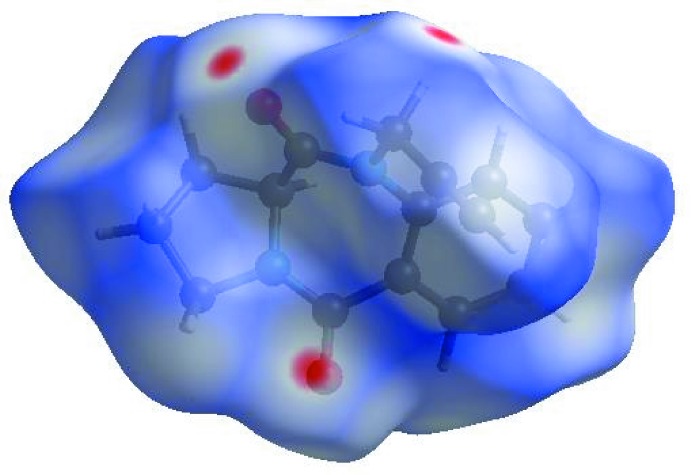
View of the three-dimensional Hirshfeld surface of the title compound plotted over *d*_norm_ in the range −0.1285 to 1.4451 a.u.

**Figure 4:**
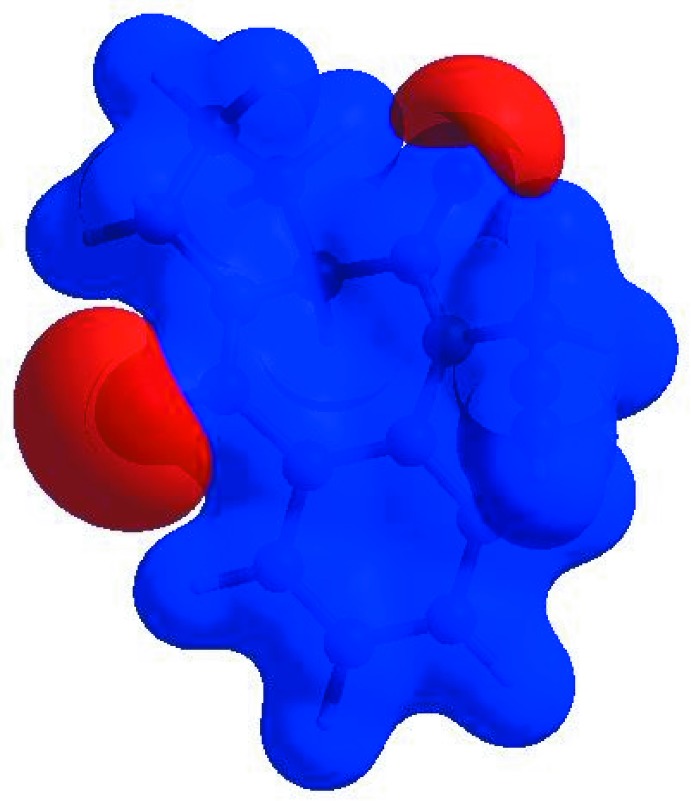
View of the three-dimensional Hirshfeld surface of the title compound plotted over electrostatic potential energy in the range −0.0500 to 0.0500 a.u. using the STO-3 G basis set at the Hartree–Fock level of theory. Hydrogen-bond donors and acceptors are shown as blue and red regions around the atoms, corresponding to positive and negative potentials, respectively.

**Figure 5:**
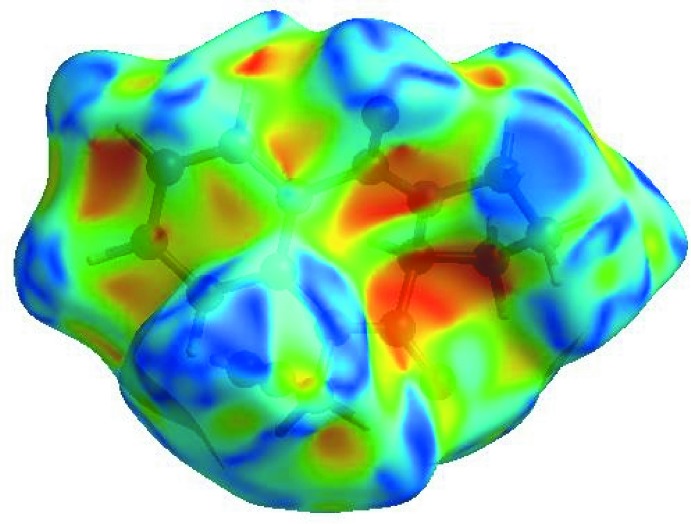
Hirshfeld surface of the title compound plotted over shape-index.

In order to visualize the inter­molecular inter­actions in the crystal of the title compound, a Hirshfeld surface (HS) analysis (Hirshfeld, 1977 ▸; Spackman & Jayatilaka, 2009 ▸) was carried out using *Crystal Explorer 17.5* (Turner *et al.*, 2017 ▸). In the HS plotted over *d*_norm_ (Fig. 3 ▸), the white surface indicates contacts with distances equal to the sum of van der Waals radii, and the red and blue colours indicate distances shorter (in close contact) or longer (distinct contact) than the van der Waals radii, respectively (Venkatesan *et al.*, 2016 ▸). The bright-red spots appearing near O1, O2 and hydrogen atom H13*A* indicate their roles as the respective donors and acceptors; they also appear as blue and red regions corresponding to positive and negative potentials on the HS mapped over electrostatic potential (Spackman *et al.*, 2008 ▸; Jayatilaka *et al.*, 2005 ▸) shown in Fig. 4 ▸. Here the blue regions indicate positive electrostatic potential (hydrogen-bond donors), while the red regions indicate negative electrostatic potential (hydrogen-bond acceptors). The shape-index of the HS is a tool to visualize the π–π stacking by the presence of adjacent red and blue triangles; if there are no adjacent red and/or blue triangles, then there are no π–π inter­actions. Fig. 5 ▸ clearly suggests that there are no π–π inter­actions in (I).

**Figure 6:**
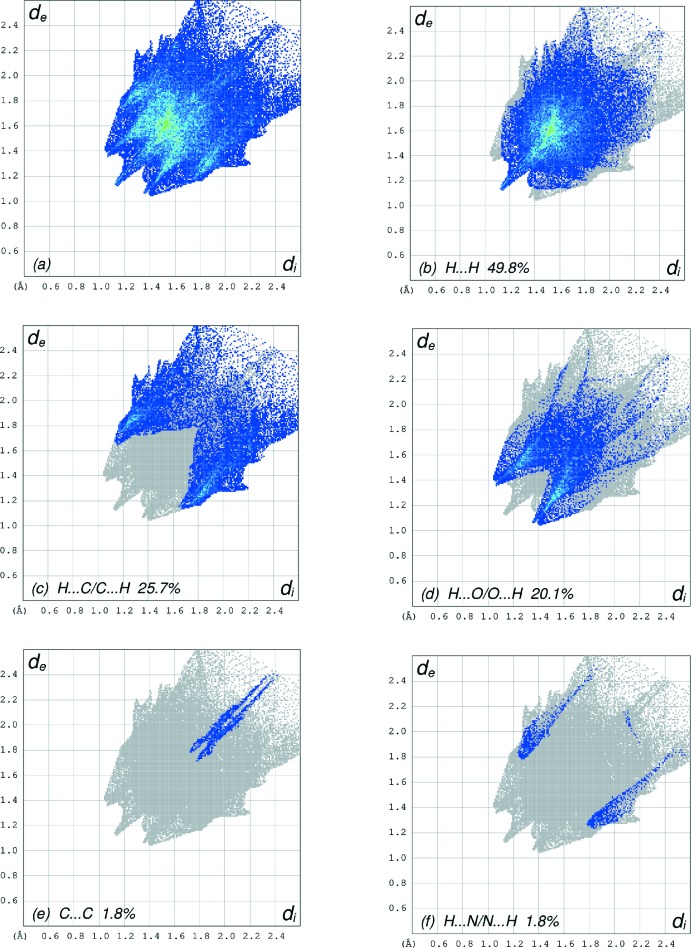
The full two-dimensional fingerprint plots for the title compound, showing (*a*) all inter­actions, and delineated into (*b*) H⋯H, (*c*) H⋯C/C⋯H, (*d*) H⋯O/O⋯H, (*e*) C⋯C and (*f*) H⋯N/N⋯H inter­actions. The *d*_i_ and *d*_e_ values are the closest inter­nal and external distances (in Å) from given points on the Hirshfeld surface contacts.

The overall two-dimensional fingerprint plot, Fig. 6 ▸ *a*, and those delineated into H⋯H, H⋯C/C⋯H, H⋯O/O⋯H, C⋯C and H⋯N/N⋯H contacts (McKinnon *et al.*, 2007 ▸) are illustrated in Fig. 6 ▸ *b*–*f*, respectively, together with their relative contributions to the Hirshfeld surface. The most important inter­action is H⋯H contributing 49.8% to the overall crystal packing, which is reflected in Fig. 6 ▸ *b* as widely scattered points of high density due to the large hydrogen content of the mol­ecule with the tip at *d*_e_ = *d*_i_ = 1.13 Å. In the absence of C—H ⋯ π inter­actions, the pairs of characteristic wings in Fig. 6 ▸ *c* arises from H⋯C/C⋯H contacts (25.7% contribution to the HS); the pair of spikes have tips at *d*_e_ + *d*_i_ = 2.80 Å. The thin and thick pairs of scattered points of wings in the fingerprint plot delineated into H⋯O/O⋯H contacts (Fig. 6 ▸ *d*, 20.1%) have a symmetrical distribution of points with the edges at *d*_e_ + *d*_i_ = 2.42 and 2.44 Å, respectively. The C⋯C contacts (Fig. 6 ▸ *e*, 1.8%) have a pliers-shaped distribution of points with the tips at *d*_e_ + *d*_i_ = 3.47 Å. Finally, the H ⋯ N/N⋯H inter­actions (1.8%) are reflected in Fig. 6 ▸ *f* as thick wings with the tips at *d*_e_ + *d*_i_ = 3.04 Å. Selected contacts are listed in Table 2 ▸.

**Figure 7:**
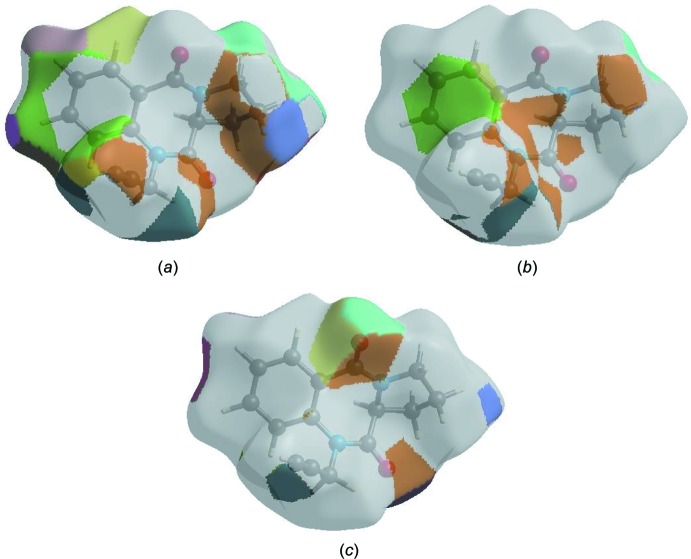
Hirshfeld surface representations with the function *d*_norm_ plotted onto the surface for (*a*) H⋯H, (*b*) H⋯C/C⋯H and (*c*) H⋯O/O⋯H inter­actions.

The Hirshfeld surface representations with the function *d*_norm_ plotted onto the surface are shown for the H⋯H, H⋯C/C⋯H and H⋯O/O⋯H inter­actions in Fig. 7 ▸ *a*–*c*, respectively.

The Hirshfeld surface analysis confirms the importance of H-atom contacts in establishing the packing. The large number of H⋯H, H⋯C/C⋯H and H⋯O/O⋯H inter­actions suggest that van der Waals inter­actions and hydrogen bonding play the major roles in the crystal packing (Hathwar *et al.*, 2015 ▸).

## Inter­action energy calculations   

The inter­molecular inter­action energies were calculated using the CE–B3LYP/6–31G(d,p) energy model in *Crystal Explorer 17.5* (Turner *et al.*, 2017 ▸), where a cluster of mol­ecules is generated by applying crystallographic symmetry operations with respect to a selected central mol­ecule within a default radius of 3.8 Å (Turner *et al.*, 2014 ▸). The total inter­molecular energy (*E*_tot_) is the sum of electrostatic (*E*_ele_), polarization (*E*_pol_), dispersion (*E*_dis_) and exchange-repulsion (*E*_rep_) energies (Turner *et al.*, 2015 ▸) with scale factors of 1.057, 0.740, 0.871 and 0.618, respectively (Mackenzie *et al.*, 2017 ▸). Hydrogen-bonding inter­action energies (in kJ mol^−1^) were calculated as −13.2 (*E*_ele_), −3.8 (*E*_pol_), −45.1 (*E*_dis_), 27.8 (*E*_rep_) and −38.8 (*E*_tot_) for C2—H2⋯O2 and −10.7 (*E*_ele_), −4.0 (*E*_pol_), −25.8 (*E*_dis_), 15.7 (*E*_rep_) and −27.1 (*E*_tot_) for C13—H13*A*⋯O1.

## DFT calculations   

**Figure 8:**
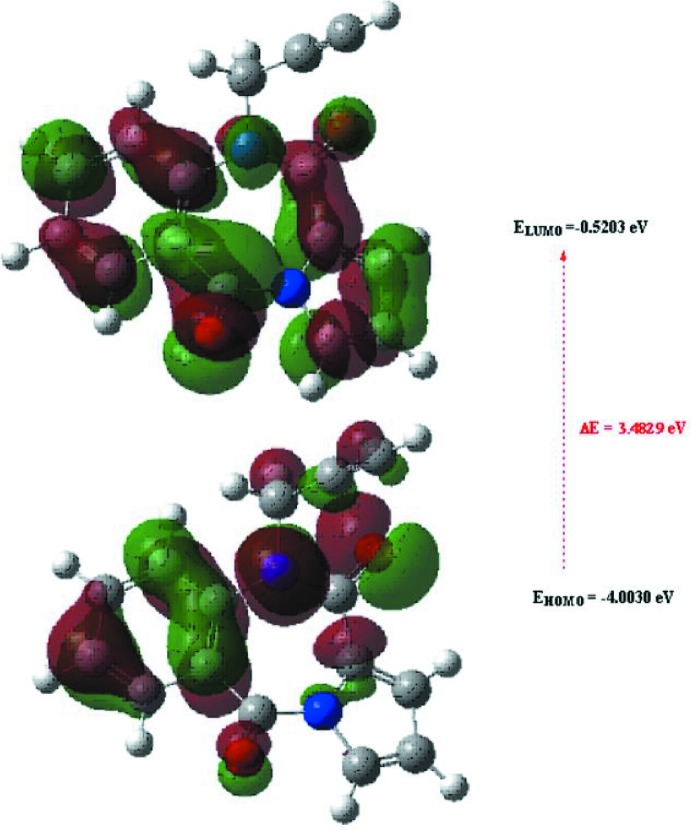
The energy band gap of the title compound.

The optimized structure of the title compound in the gas phase was generated theoretically *via* density functional theory (DFT) using standard B3LYP functional and 6–311 G(d,p) basis-set calculations (Becke, 1993 ▸) as implemented in *GAUSSIAN 09* (Frisch *et al.*, 2009 ▸). The theoretical and experimental results were in good agreement (Table 3 ▸). The highest-occupied mol­ecular orbital (HOMO), acting as an electron donor, and the lowest-unoccupied mol­ecular orbital (LUMO), acting as an electron acceptor, are very important parameters for quantum chemistry. When the energy gap is small, the mol­ecule is highly polarizable and has high chemical reactivity. The DFT calculations provide some important information on the reactivity and site selectivity of the mol­ecular framework. *E*_HOMO_ and *E*_LUMO_ clarify the inevitable charge-exchange collaboration inside the studied material, and are given in Table 4 ▸ along with the electronegativity (χ), hardness (η), potential (μ), electrophilicity (ω) and softness (*σ*). The significance of η and *σ* is to evaluate both the reactivity and stability. The electron transition from the HOMO to the LUMO energy level is shown in Fig. 8 ▸. The HOMO and LUMO are localized in the plane extending from the whole 10-propargyl­pyrrolo­[2,1-*c*][1,4]benzodiazepine-5,11-dione ring. The energy band gap [Δ*E* = *E*_LUMO_ − *E*_HOMO_] of the mol­ecule is 3.4829 eV, and the frontier mol­ecular orbital energies, *E*_HOMO_ and *E*_LUMO_ are −4.0030 and −0.5203 eV, respectively.

## Database survey   

A alkyl­ated analogue has been reported, *viz*. 10-allyl-2,3-di­hydro-1*H*-pyrrolo­[2,1-*c*][1,4]benzodiazepine-5,11(10*H*,11a*H*)-dione (Benzeid *et al.*, 2009*a* ▸), as well as three similar structures, 2-hy­droxy-10-propargyl­pyrrolo­[2,1-*c*][1,4]benzodiazepine-5,11-dione monohydrate (Ourahou *et al.* 2010 ▸), *rac*-9,10-dimeth­oxy-3-methyl-6-phenyl-7,7adi­hydro­benzo[*b*]benzo[4,5]iso­thia­zolo[2,3-*d*][1,4]diazepine 12,12-dioxide (Bassin *et al.*, 2011 ▸) and (*S*)-2,3,5,10,11,11a-hexa­hydro-1*H*-pyrrolo­[2,1-*c*][1,4]benzodiazepine-3,11-dione (Cheng *et al.* 2007 ▸).

## Synthesis and crystallization   

The synthesis of pyrrolo­benzodiazepine is a simple condensation of isatoic anhydride on l-proline. Pyrrolo­[2,1-*c*][1,4]benzodiazepine-5,11-dione (2.15 mmol), propargyl bromide (2.15 mmol) and potassium carbonate (4.3 mmol) along with a catalytic amount of tetra-*n*-butyl ammonium bromide were stirred in *N*,*N*-di­methyl­formamide (20 ml) for 72 h. The solid material was removed by filtration and the solvent evaporated under vacuum. The residue was separated by chromatography on silica gel with an *n*-hexa­ne–ethyl acetate (1:9) solvent system. The title compound was obtained as colourless crystals in 70% yield upon evaporation of the solvent.

## Refinement   

Crystal data, data collection and structure refinement details are summarized in Table 5 ▸. The C-bound H atoms were positioned geometrically, with C—H = 0.93 Å (for aromatic and propagyl moiety’s H atoms), 0.98 Å (for methine H atom) and 0.97 Å (for methyl­ene H atoms), and constrained to ride on their parent atoms, with *U*_iso_(H) = 1.*U*_eq_(C).

## Supplementary Material

Crystal structure: contains datablock(s) I, global. DOI: 10.1107/S2056989020002698/lh5947sup1.cif

Structure factors: contains datablock(s) I. DOI: 10.1107/S2056989020002698/lh5947Isup2.hkl

Supporting information file. DOI: 10.1107/S2056989020002698/lh5947Isup3.cdx

Supporting information file. DOI: 10.1107/S2056989020002698/lh5947Isup4.cml

CCDC reference: 1986475

Additional supporting information:  crystallographic information; 3D view; checkCIF report

## Figures and Tables

**Table 1:** Hydrogen-bond geometry (Å, °)

*D*—H⋯*A*	*D*—H	H⋯*A*	*D*⋯*A*	*D*—H⋯*A*
C2—H2⋯O2^vii^	0.93	2.53	3.252 (2)	135
C13—H13*A*⋯O1^viii^	0.97	2.54	3.395 (3)	147

Symmetry codes: (vii) 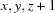; (viii) 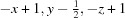.

**Table 2:** Selected interatomic distances (Å)

O1⋯C15^i^	3.273 (4)	O2⋯H4^ii^	2.69
O1⋯C13^ii^	3.395 (3)	N1⋯N2	2.898 (2)
O2⋯C2^iii^	3.252 (2)	C2⋯C10^v^	3.558 (3)
O2⋯C11	3.303 (3)	C4⋯C12^vi^	3.552 (4)
O2⋯C4^ii^	3.397 (3)	C5⋯C14	3.090 (4)
O1⋯H8^iv^	2.82	C7⋯C15^i^	3.512 (4)
O1⋯H12*A* ^iv^	2.76	C1⋯H8	2.69
O1⋯H2	2.63	C3⋯H12*A* ^vi^	2.90
O1⋯H10*A*	2.73	C3⋯H11*B* ^v^	2.87
O1⋯H15^i^	2.81	C5⋯H13*A*	2.86
O1⋯H13*A* ^ii^	2.54	C6⋯H8	2.69
O2⋯H2^iii^	2.53	C7⋯H13*A* ^ii^	2.93
O2⋯H12*B*	2.45	C13⋯H5	2.66
O2⋯H13*B*	2.32	C14⋯H5	2.92
O2⋯H11*A*	2.89	H5⋯H13*A*	2.32

Symmetry codes: (i) 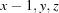; (ii) 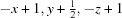; (iii) 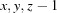; (iv) 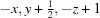; (v) 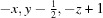; (vi) 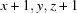.

**Table 3:** Comparison of the selected (X-ray and DFT) geometric data (Å, °)

Bonds/angles	X-ray	B3LYP/6–311G(d,p)
O1—C7	1.231 (2)	1.30064
O2—C9	1.222 (2)	1.30459
N1—C7	1.337 (2)	1.44900
N1—C8	1.474 (2)	1.42892
N1—C10	1.476 (2)	1.41852
N2—C6	1.429 (2)	1.45461
N2—C9	1.362 (2)	1.45679
N2—C13	1.478 (2)	1.48990
		
C7—N1—C8	124.64 (14)	125.54242
C7—N1—C10	122.81 (16)	120.48706
C8—N1—C10	112.28 (14)	111.27162
C6—N2—C13	118.67 (14)	116.39016
C9—N2—C6	123.48 (14)	122.08303
C9—N2—C13	116.98 (15)	113.69042
C1—C6—N2	122.46 (14)	120.60573
C5—C6—N2	118.13 (15)	117.33963

**Table 4:** Calculated energies

Mol­ecular Energy (a.u.) (eV)	Compound (I)
Total Energy, *TE* (eV)	−22499
*E*_HOMO_ (eV)	−4.0030
*E*_LUMO_ (eV)	−0.5203
Gap *ΔE* (eV)	3.4829
Dipole moment, *μ* (Debye)	2.2189
Ionization potential, *I* (eV)	4.0030
Electron affinity, *A*	0.5203
Electronegativity, *χ*	2.2617
Hardness, *η*	1.7414
Electrophilicity index, *ω*	1.4687
Softness, *σ*	0.5742
Fraction of electron transferred, *ΔN*	1.3605

**Table 5:** Experimental details

Crystal data	
Chemical formula	C_15_H_14_N_2_O_2_
*M*_r_	254.28
Crystal system, space group	Monoclinic, *P*2_1_
Temperature (K)	299
*a*, *b*, *c* (Å)	8.4959 (2), 9.6479 (2), 8.7619 (2)
β (°)	116.921 (1)
*V* (Å^3^)	640.36 (3)
*Z*	2
Radiation type	Mo *K*α
μ (mm^−1^)	0.09
Crystal size (mm)	0.39 × 0.37 × 0.16

Data collection	
Diffractometer	Bruker APEXII CCD
Absorption correction	Multi-scan (*SADABS*; Bruker, 2013 ▸)
*T*_min_, *T*_max_	0.684, 0.746
No. of measured, independent and observed [*I* > 2σ(*I*)] reflections	12206, 3821, 3349
*R*_int_	0.024
(sin θ/λ)_max_ (Å^−1^)	0.714

Refinement	
*R*[*F* ^2^ > 2σ(*F* ^2^)], *wR*(*F* ^2^), *S*	0.039, 0.103, 1.06
No. of reflections	3821
No. of parameters	172
No. of restraints	1
H-atom treatment	H-atom parameters constrained
Δρ_max_, Δρ_min_ (e Å^−3^)	0.20, −0.15
Absolute structure	Flack *x* determined using 1347 quotients [(*I* ^+^)−(*I* ^−^)]/[(*I* ^+^)+(*I* ^−^)] (Parsons *et al.*, 2013 ▸)
Absolute structure parameter	−0.4 (3)

Computer programs: *APEX3* and *SAINT* (Bruker, 2013 ▸), *SHELXT* (Sheldrick, 2015*a* ▸), *SHELXL* (Sheldrick, 2015*b* ▸) and *OLEX2* (Dolomanov *et al.*, 2009 ▸).

## supplementary crystallographic information

### Crystal data

**Table:**

C_15_H_14_N_2_O_2_	*F*(000) = 268
*M**_r_* = 254.28	*D*_x_ = 1.319 Mg m^−^^3^
Monoclinic, *P*2_1_	Mo *K*α radiation, λ = 0.71073 Å
*a* = 8.4959 (2) Å	Cell parameters from 6478 reflections
*b* = 9.6479 (2) Å	θ = 2.6–28.5°
*c* = 8.7619 (2) Å	µ = 0.09 mm^−^^1^
β = 116.921 (1)°	*T* = 299 K
*V* = 640.36 (3) Å^3^	Plate, clear light colourless
*Z* = 2	0.39 × 0.37 × 0.16 mm

### Data collection

**Table:**

Bruker APEXII CCD diffractometer	3349 reflections with *I* > 2σ(*I*)
φ and ω scans	*R*_int_ = 0.024
Absorption correction: multi-scan (SADABS; Bruker, 2013)	θ_max_ = 30.5°, θ_min_ = 2.6°
*T*_min_ = 0.684, *T*_max_ = 0.746	*h* = −12→12
12206 measured reflections	*k* = −13→13
3821 independent reflections	*l* = −12→12

### Refinement

**Table:**

Refinement on *F*^2^	Hydrogen site location: inferred from neighbouring sites
Least-squares matrix: full	H-atom parameters constrained
*R*[*F*^2^ > 2σ(*F*^2^)] = 0.039	*w* = 1/[σ^2^(*F*_o_^2^) + (0.0554*P*)^2^ + 0.0351*P*] where *P* = (*F*_o_^2^ + 2*F*_c_^2^)/3
*wR*(*F*^2^) = 0.103	(Δ/σ)_max_ < 0.001
*S* = 1.06	Δρ_max_ = 0.20 e Å^−^^3^
3821 reflections	Δρ_min_ = −0.15 e Å^−^^3^
172 parameters	Absolute structure: Flack *x* determined using 1347 quotients [(*I*^+^)-(*I*^-^)]/[(*I*^+^)+(*I*^-^)] (Parsons *et al.*, 2013)
1 restraint	Absolute structure parameter: −0.4 (3)
Primary atom site location: dual	

### Special details

**Table:**

Geometry. All esds (except the esd in the dihedral angle between two l.s. planes) are estimated using the full covariance matrix. The cell esds are taken into account individually in the estimation of esds in distances, angles and torsion angles; correlations between esds in cell parameters are only used when they are defined by crystal symmetry. An approximate (isotropic) treatment of cell esds is used for estimating esds involving l.s. planes.

### Fractional atomic coordinates and isotropic or equivalent isotropic displacement parameters (Å^2^)

**Table:**

	*x*	*y*	*z*	*U*_iso_*/*U*_eq_	
O1	0.1993 (2)	0.71379 (15)	0.67243 (19)	0.0515 (4)	
O2	0.2576 (2)	0.4767 (2)	0.15885 (17)	0.0621 (4)	
N1	0.10489 (19)	0.59648 (15)	0.42473 (19)	0.0384 (3)	
N2	0.41816 (19)	0.46005 (16)	0.44625 (18)	0.0389 (3)	
C1	0.3344 (2)	0.49614 (17)	0.68011 (19)	0.0342 (3)	
C2	0.3604 (2)	0.4606 (2)	0.8441 (2)	0.0441 (4)	
H2	0.297976	0.507584	0.892040	0.053*	
C3	0.4764 (3)	0.3576 (2)	0.9365 (2)	0.0518 (5)	
H3	0.491032	0.334562	1.045179	0.062*	
C4	0.5709 (3)	0.2888 (2)	0.8670 (3)	0.0525 (5)	
H4	0.650555	0.219823	0.929588	0.063*	
C5	0.5477 (3)	0.3218 (2)	0.7046 (3)	0.0466 (4)	
H5	0.611399	0.274396	0.658401	0.056*	
C6	0.4297 (2)	0.42536 (17)	0.6096 (2)	0.0347 (3)	
C7	0.2084 (2)	0.61152 (17)	0.5927 (2)	0.0360 (3)	
C8	0.0974 (2)	0.47097 (19)	0.3258 (2)	0.0383 (3)	
H8	0.088877	0.387499	0.385319	0.046*	
C9	0.2629 (2)	0.4673 (2)	0.3002 (2)	0.0396 (4)	
C10	−0.0288 (3)	0.7001 (2)	0.3214 (3)	0.0540 (5)	
H10A	0.021993	0.792198	0.337758	0.065*	
H10B	−0.126879	0.701151	0.349469	0.065*	
C11	−0.0863 (3)	0.6494 (3)	0.1404 (3)	0.0668 (7)	
H11A	−0.009804	0.685371	0.094619	0.080*	
H11B	−0.206984	0.676803	0.065910	0.080*	
C12	−0.0705 (3)	0.4919 (3)	0.1595 (3)	0.0561 (5)	
H12A	−0.171813	0.452660	0.167103	0.067*	
H12B	−0.060151	0.449916	0.063901	0.067*	
C13	0.5822 (3)	0.4646 (3)	0.4271 (3)	0.0522 (5)	
H13A	0.618467	0.370691	0.419118	0.063*	
H13B	0.558798	0.512036	0.321305	0.063*	
C14	0.7252 (3)	0.5347 (3)	0.5684 (3)	0.0559 (5)	
C15	0.8456 (4)	0.5887 (4)	0.6817 (4)	0.0771 (8)	
H15	0.940820	0.631390	0.771234	0.092*	

### Atomic displacement parameters (Å^2^)

**Table:**

	*U*^11^	*U*^22^	*U*^33^	*U*^12^	*U*^13^	*U*^23^
O1	0.0594 (8)	0.0445 (7)	0.0560 (9)	−0.0014 (7)	0.0310 (7)	−0.0151 (6)
O2	0.0678 (9)	0.0904 (12)	0.0351 (7)	0.0028 (9)	0.0293 (6)	−0.0011 (8)
N1	0.0379 (7)	0.0392 (8)	0.0384 (8)	0.0031 (6)	0.0175 (6)	−0.0009 (6)
N2	0.0431 (7)	0.0439 (8)	0.0358 (7)	0.0055 (7)	0.0233 (6)	0.0027 (6)
C1	0.0366 (8)	0.0360 (8)	0.0303 (7)	−0.0079 (6)	0.0155 (6)	−0.0054 (6)
C2	0.0487 (9)	0.0538 (11)	0.0312 (8)	−0.0131 (9)	0.0191 (7)	−0.0088 (8)
C3	0.0566 (11)	0.0604 (12)	0.0291 (8)	−0.0183 (10)	0.0112 (8)	0.0028 (8)
C4	0.0526 (11)	0.0467 (11)	0.0432 (10)	−0.0022 (9)	0.0085 (9)	0.0108 (8)
C5	0.0476 (10)	0.0408 (10)	0.0479 (10)	0.0048 (8)	0.0186 (8)	0.0039 (8)
C6	0.0380 (8)	0.0349 (8)	0.0314 (8)	−0.0015 (6)	0.0160 (7)	0.0001 (6)
C7	0.0379 (8)	0.0362 (8)	0.0400 (9)	−0.0062 (6)	0.0230 (7)	−0.0055 (6)
C8	0.0425 (8)	0.0395 (8)	0.0326 (7)	−0.0066 (7)	0.0166 (6)	−0.0036 (7)
C9	0.0507 (9)	0.0383 (8)	0.0340 (8)	0.0012 (8)	0.0228 (7)	−0.0013 (7)
C10	0.0473 (10)	0.0560 (12)	0.0551 (12)	0.0131 (9)	0.0201 (9)	0.0082 (9)
C11	0.0597 (13)	0.0829 (17)	0.0490 (12)	0.0237 (13)	0.0169 (10)	0.0158 (12)
C12	0.0469 (10)	0.0737 (15)	0.0373 (9)	−0.0080 (10)	0.0099 (8)	−0.0042 (9)
C13	0.0510 (10)	0.0661 (13)	0.0520 (11)	0.0141 (10)	0.0343 (9)	0.0040 (10)
C14	0.0480 (11)	0.0657 (13)	0.0659 (14)	0.0053 (10)	0.0364 (11)	0.0103 (11)
C15	0.0588 (14)	0.093 (2)	0.0800 (19)	−0.0120 (14)	0.0319 (13)	0.0044 (15)

### Geometric parameters (Å, º)

**Table:**

O1—C7	1.231 (2)	C5—C6	1.393 (2)
O2—C9	1.222 (2)	C8—H8	0.9800
N1—C7	1.337 (2)	C8—C9	1.521 (3)
N1—C8	1.474 (2)	C8—C12	1.522 (3)
N1—C10	1.476 (2)	C10—H10A	0.9700
N2—C6	1.429 (2)	C10—H10B	0.9700
N2—C9	1.362 (2)	C10—C11	1.513 (3)
N2—C13	1.478 (2)	C11—H11A	0.9700
C1—C2	1.394 (2)	C11—H11B	0.9700
C1—C6	1.399 (2)	C11—C12	1.527 (4)
C1—C7	1.493 (2)	C12—H12A	0.9700
C2—H2	0.9300	C12—H12B	0.9700
C2—C3	1.376 (3)	C13—H13A	0.9700
C3—H3	0.9300	C13—H13B	0.9700
C3—C4	1.378 (3)	C13—C14	1.450 (3)
C4—H4	0.9300	C14—C15	1.176 (4)
C4—C5	1.382 (3)	C15—H15	0.9300
C5—H5	0.9300		
			
O1···C15^i^	3.273 (4)	O2···H4^ii^	2.69
O1···C13^ii^	3.395 (3)	N1···N2	2.898 (2)
O2···C2^iii^	3.252 (2)	C2···C10^v^	3.558 (3)
O2···C11	3.303 (3)	C4···C12^vi^	3.552 (4)
O2···C4^ii^	3.397 (3)	C5···C14	3.090 (4)
O1···H8^iv^	2.82	C7···C15^i^	3.512 (4)
O1···H12A^iv^	2.76	C1···H8	2.69
O1···H2	2.63	C3···H12A^vi^	2.90
O1···H10A	2.73	C3···H11B^v^	2.87
O1···H15^i^	2.81	C5···H13A	2.86
O1···H13A^ii^	2.54	C6···H8	2.69
O2···H2^iii^	2.53	C7···H13A^ii^	2.93
O2···H12B	2.45	C13···H5	2.66
O2···H13B	2.32	C14···H5	2.92
O2···H11A	2.89	H5···H13A	2.32
			
C7—N1—C8	124.64 (14)	C9—C8—C12	112.98 (15)
C7—N1—C10	122.81 (16)	C12—C8—H8	110.8
C8—N1—C10	112.28 (14)	O2—C9—N2	122.16 (17)
C6—N2—C13	118.67 (14)	O2—C9—C8	122.34 (16)
C9—N2—C6	123.48 (14)	N2—C9—C8	115.42 (14)
C9—N2—C13	116.98 (15)	N1—C10—H10A	111.2
C2—C1—C6	118.81 (16)	N1—C10—H10B	111.2
C2—C1—C7	117.02 (15)	N1—C10—C11	102.59 (18)
C6—C1—C7	124.15 (14)	H10A—C10—H10B	109.2
C1—C2—H2	119.3	C11—C10—H10A	111.2
C3—C2—C1	121.41 (18)	C11—C10—H10B	111.2
C3—C2—H2	119.3	C10—C11—H11A	111.0
C2—C3—H3	120.2	C10—C11—H11B	111.0
C2—C3—C4	119.60 (18)	C10—C11—C12	103.65 (19)
C4—C3—H3	120.2	H11A—C11—H11B	109.0
C3—C4—H4	119.9	C12—C11—H11A	111.0
C3—C4—C5	120.23 (19)	C12—C11—H11B	111.0
C5—C4—H4	119.9	C8—C12—C11	103.60 (18)
C4—C5—H5	119.7	C8—C12—H12A	111.0
C4—C5—C6	120.63 (19)	C8—C12—H12B	111.0
C6—C5—H5	119.7	C11—C12—H12A	111.0
C1—C6—N2	122.46 (14)	C11—C12—H12B	111.0
C5—C6—N2	118.13 (15)	H12A—C12—H12B	109.0
C5—C6—C1	119.32 (15)	N2—C13—H13A	109.1
O1—C7—N1	122.16 (17)	N2—C13—H13B	109.1
O1—C7—C1	121.40 (16)	H13A—C13—H13B	107.8
N1—C7—C1	116.43 (14)	C14—C13—N2	112.66 (17)
N1—C8—H8	110.8	C14—C13—H13A	109.1
N1—C8—C9	108.01 (14)	C14—C13—H13B	109.1
N1—C8—C12	103.06 (16)	C15—C14—C13	177.4 (3)
C9—C8—H8	110.8	C14—C15—H15	180.0

Symmetry codes: (i) *x*−1, *y*, *z*; (ii) −*x*+1, *y*+1/2, −*z*+1; (iii) *x*, *y*, *z*−1; (iv) −*x*, *y*+1/2, −*z*+1; (v) −*x*, *y*−1/2, −*z*+1; (vi) *x*+1, *y*, *z*+1.

### Hydrogen-bond geometry (Å, º)

**Table:**

*D*—H···*A*	*D*—H	H···*A*	*D*···*A*	*D*—H···*A*
C2—H2···O2^vii^	0.93	2.53	3.252 (2)	135
C13—H13*A*···O1^viii^	0.97	2.54	3.395 (3)	147

Symmetry codes: (vii) *x*, *y*, *z*+1; (viii) −*x*+1, *y*−1/2, −*z*+1.
